# Transthoracic echocardiography-monitored CO_2_-insufflation esophageal endoscopy for diagnosis of Atrioesophageal fistula and prevention of iatrogenic air embolism: a case report

**DOI:** 10.1186/s12872-020-01503-3

**Published:** 2020-05-12

**Authors:** Bing Rong, Xiquan Zhang, Hui Tian, Hongyu Zhang, Ning Zhong, Jingquan Zhong

**Affiliations:** 1 The Key Laboratory of Cardiovascular Remodeling and Function Research, Chinese Ministry of Education, Chinese National Health Commission, and Chinese Academy of Medical Sciences, The State and Shandong Province Joint Key Laboratory of Translational Cardiovascular Medicine, Qilu Hospital of Shandong University, 107 Wenhuaxi Road, Jinan, 250012 China; 2 Department of Geriatric Medicine, Qilu Hospital of Shandong University, 107 Wenhuaxi Road, Jinan, 250012 China; 3 Department of Cadiovascular Surgery, Qilu Hospital of Shandong University, 107 Wenhuaxi Road, Jinan, 250012 China; 4 Department of Thoracic Surgery, Qilu Hospital of Shandong University, 107 Wenhuaxi Road, Jinan, 250012 China; 5 Department of Gastroenterology, Qilu Hospital of Shandong University, 107 Wenhuaxi Road, Jinan, 250012 China

**Keywords:** Atrial fibrillation, Atrioesophageal fistula, Esophageal endoscopy, Carbon dioxide

## Abstract

**Background:**

Atrioesophageal fistula (AEF) is the most fatal complication associated with catheter ablation for atrial fibrillation and cannot be easily detected when thoracic contrast-enhanced computed tomography (CT) is normal.

**Case presentation:**

In this report, we described a diagnostic tool for detecting AEF with doubtful chest CT in which we introduced CO_2_-insufflation esophageal endoscopy with transthoracic echocardiography monitoring. Using this modified esophageal endoscopy, AEF was established due to the presence of both esophageal lesions and bubbles into the left atrium. That way, our patient accepted to be operated in time with good clinical prognosis.

**Conclusions:**

This modified esophageal endoscopy is an alternative tool for early detection of AEF when normal or doubtful CT findings present.

## Background

Atrioesophageal fistula is a rare but the most fatal complication associated with catheter ablation in atrial fibrillation (AF) (0.03–0.08%) [1]. Chest computed tomography (CT) is recommended for detecting AEF, with a high prevalence of imaging abnormalities (80–90%), while direct imaging abnormalities are seen in just 30–40% [2]. About 10–20% of cases, especially during the early phase of AEF, have no CT abnormalities. Repeat CT may take at least 4 days. Thus, it may result in poor prognosis [2]. An alternative strategy, according to an expert consensus statement, is the use of carbon dioxide (CO_2_)-insufflation esophageal endoscopy [3]. We report a case using transthoracic echocardiography (TTE) monitoring during CO_2_-insufflation esophageal endoscopy as a modality for early detection of AEF.

## Case presentation

**Fig. 1:**
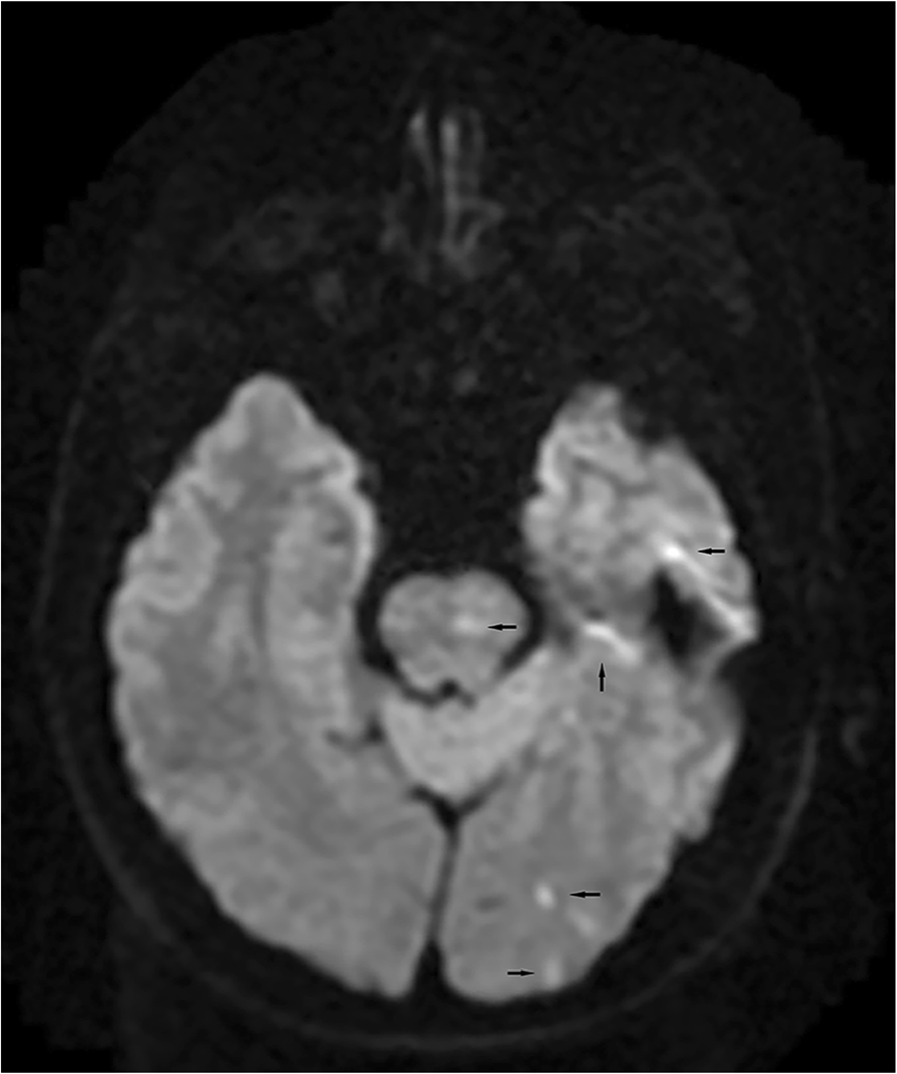
Brain magnetic resonance imaging (diffusion-weighted imaging sequence): multiple acute ischemic lesions (black arrows)

**Fig. 2:**
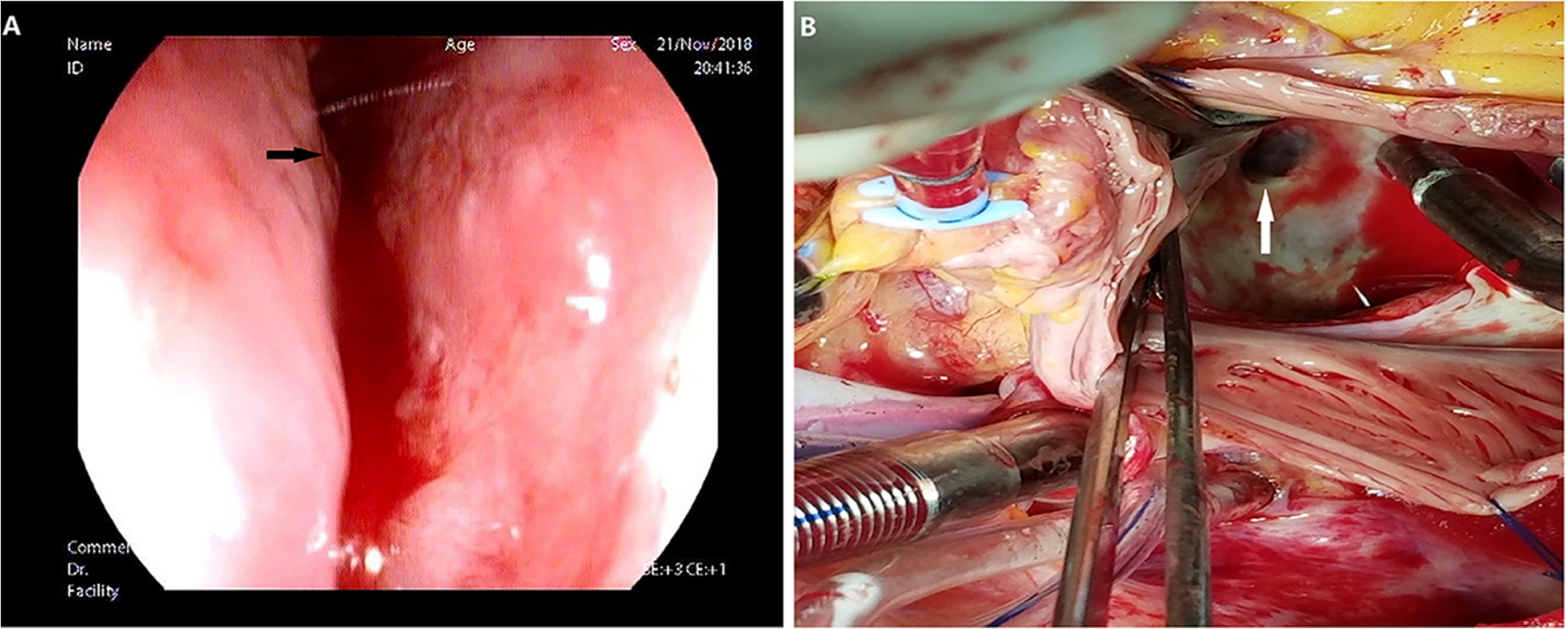
Esophageal endoscopy: **a** 5-mm fistula with active bleeding localized on the anterior esophageal wall (black arrow); Intraoperative photograph: **b** 10-mm atrial defect near the left inferior pulmonary vein (white arrow)

A 57-year-old male patient underwent radiofrequency ablation for persistent atrial fibrillation 40 days prior, and he presented with transient numbness and weakness in his left extremity and new-onset hematemesis and fever (38.9 °C). Laboratory findings showed elevated white blood cell count (13,100/mm^3^). Brain magnetic resonance imaging (MRI) revealed multiple areas of acute cerebral infarction (Fig. 1). Chest contrast-enhanced CT showed a suspected hypodensity region in the posterior aspect of the left atrium (Supplementary figure S1). TTE excluded any heart valve disease, left ventricle contractility impairment and intracardiac thrombus or vegetation. AEF was suspected due to his presenting symptoms and MRI findings. While CT scan didn’t present the obvious abnormality, to confirm this suspicion, CO_2_ insufflation was administered during esophageal endoscopy. TTE was constantly used to monitor intracardiac bubbles to avoid an iatrogenic air embolism due to uncontrolled introduction of CO_2_ into left atrium. During the proposed procedure, if bubbles were visualized on TTE, the examination would be stopped immediately and the remaining gas would be pumped out. At 32 cm from the incisors, a 5-mm fistula with active bleeding was seen on the anterior esophageal wall (Fig. 2a). At the end of the examination, bubbles were suddenly seen in the left atrium, with no change in the electrocardiogram and no occurrence of cardiac or neurological symptoms. Thus, AEF was confirmed 3 h after admission. Emergency surgery was performed. During surgery, a 10-mm atrial defect near the left inferior pulmonary vein was repaired using a bovine pericardial patch (Fig. 2b). A 5-mm perforation on the anterior esophageal wall was directly sewn. On postoperative day 7, a cine esophagogram with oral contrast showed no leakage. On postoperative day 30, the proposed esophageal endoscopy found the fistula healing, and the patient was discharged with a normal diet, complete neurologic recovery, and no AF episodes.

## Discussion & Conclusion

A case of cardiac ischemia and the necessity for cardiopulmonary resuscitation during CO_2_-insufflation esophageal endoscopy was reported before. This indicates the potential risk of an iatrogenic air embolism when CO_2_ is uncontrollably introduced [4]. We suggested a modified CO_2_-insufflation esophageal endoscopy technique using TTE monitoring to safely and directly diagnose AEF early. We propose it as an alternative modality when chest CT is normal. It is reported that a 5-mL intracoronary CO_2_ injection had a profound influence on left ventricular function in swine [5]. Therefore, during CO2-insufflation esophageal endoscopy, the CO_2_ amount administered must be carefully controlled. Contrast-enhanced TTE is a safe and widely used method to detect patent foramen ovale because of high sensitivity of TTE to air signal. The contrast agent is a mixture of 9-mL saline and 1-mL air [6]. That means that few amount of microembolic air is safe. The modified modality employed in the present case promises of lower risk of iatrogenic air embolism as few CO_2_ is introduced into systemic circulation thanks to simultaneous TTE monitoring and pumping out of the remaining CO_2_. Therefore with this modality, AEF can be early diagnosed if esophageal lesions are uncovered or bubbles appear in the left heart. Due to lack of publication and series of cases, the reliability of this method needs further investigation.

In conclusion, this TTE-monitored CO_2_-insufflation esophageal endoscopy is an alternative modality for early confirmation of AEF when there is a high level of suspicion and a normal chest CT result.

## Supplementary information

**Additional file 1: Supplementary Figure S1.** Chest enhanced computed tomography, a suspected hypodensity region in the posterior aspect of the left atrium, no extravasation of contrast, no free air in mediastinum, pericardium or left heart.

## Data Availability

Not applicable.

## Abbreviations

AEF: Atrioesophageal fistula
AF: Atrial fibrillation
CT: Computed tomography
CO_2_: Carbon dioxide
TTE: Transthoracic echocardiography
MRI: Magnetic resonance imaging
