# Supplementary figures and images for: Transthoracic echocardiography-monitored CO2-insufflation esophageal endoscopy for diagnosis of Atrioesophageal fistula and prevention of iatrogenic air embolism: a case report
Source: BMC Cardiovasc Disord. 2020 May 12;20:219. doi: 10.1186/s12872-020-01503-3 (PMC7216549; doi:10.1186/s12872-020-01503-3)

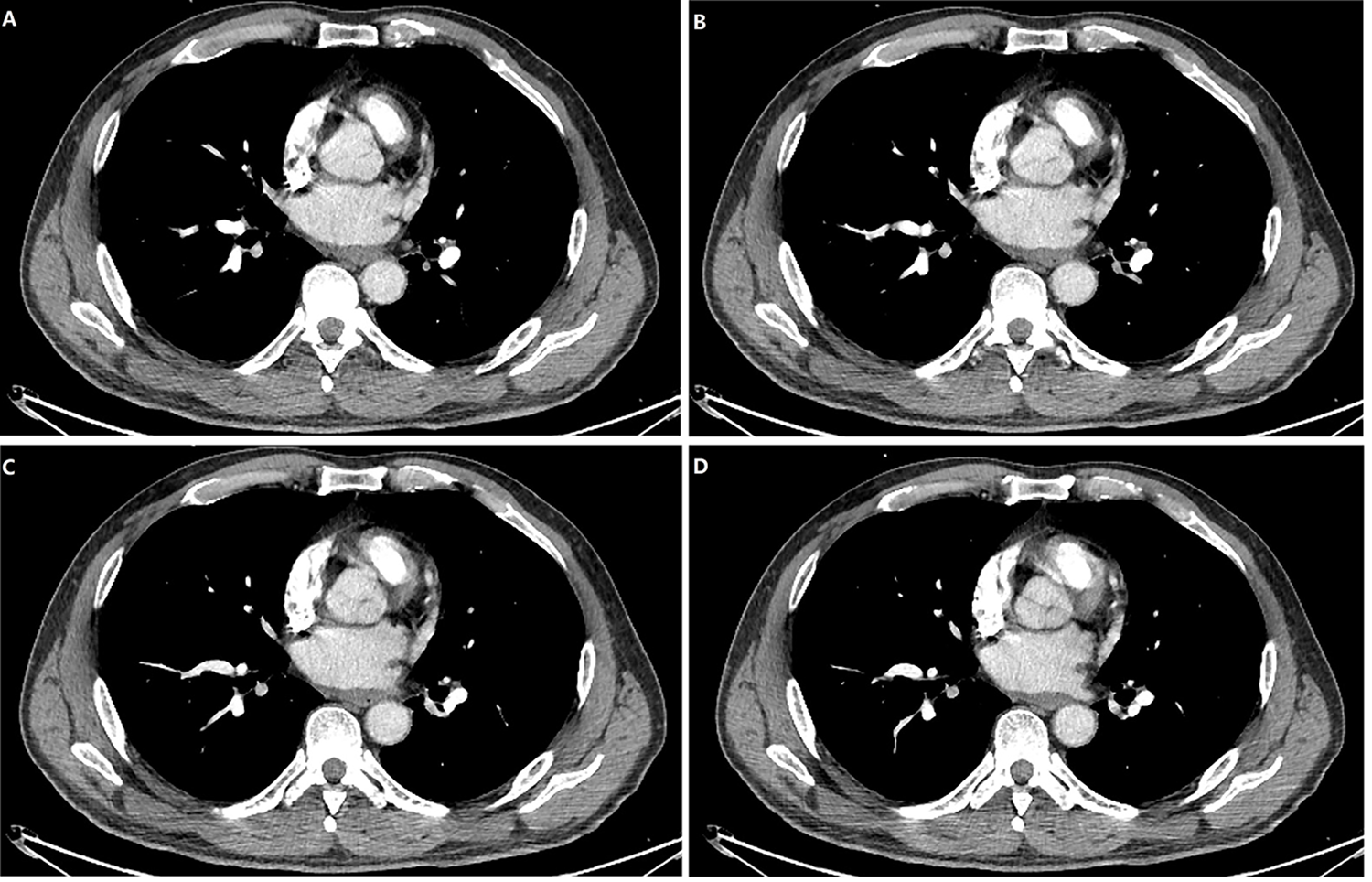

Supplement: Supplementary file 1 — Additional file 1: Supplementary Figure S1. Chest enhanced computed tomography, a suspected hypodensity region in the posterior aspect of the left atrium, no extravasation of contrast, no free air in mediastinum, pericardium or left heart. [file 12872_2020_1503_MOESM1_ESM.tif]
